# Effects of differently shaped TiO_2_NPs (nanospheres, nanorods and nanowires) on the *in vitro* model (Caco-2/HT29) of the intestinal barrier

**DOI:** 10.1186/s12989-018-0269-x

**Published:** 2018-08-07

**Authors:** Alba García-Rodríguez, Laura Vila, Constanza Cortés, Alba Hernández, Ricard Marcos

**Affiliations:** 1 Grup de Mutagènesi, Departament de Genètica i de Microbiologia, Facultat de Biociències, Universitat Autònoma de Barcelona, Edifici Cn, Campus de Bellaterra, 08193 Cerdanyola del Vallès, Barcelona Spain; 2 CIBER Epidemiología y Salud Pública, ISCIII, Madrid, Spain

**Keywords:** TiO_2_NPs nanospheres, TiO_2_NPs nanorods, TiO_2_NPs nanowires, Intestinal barrier, *In vitro*

## Abstract

**Background:**

The biological effects of nanoparticles depend on several characteristics such as size and shape that must be taken into account in any type of assessment. The increased use of titanium dioxide nanoparticles (TiO_2_NPs) for industrial applications, and specifically as a food additive, demands a deep assessment of their potential risk for humans, including their abilities to cross biological barriers.

**Methods:**

We have investigated the interaction of three differently shaped TiO_2_NPs (nanospheres, nanorods and nanowires) in an *in vitro* model of the intestinal barrier, where the coculture of Caco-2/HT29 cells confers inherent intestinal epithelium characteristics to the model (i.e. mucus secretion, brush border, tight junctions, etc.).

**Results:**

Adverse effects in the intestinal epithelium were detected by studying the barrier’s integrity (TEER), permeability (LY) and changes in the gene expression of selected specific markers. Using Laser Scanning Confocal Microscopy, we detected a different behaviour in the bio-adhesion and biodistribution of each of the TiO_2_NPs. Moreover, we were able to specifically localize each type of TiO_2_NPs inside the cells. Interestingly, general DNA damage, but not oxidative DNA damage effects, were detected by using the FPG version of the comet assay.

**Conclusions:**

Results indicate different interactions and cellular responses related to differently shaped TiO_2_NPs, nanowires showing the most harmful effects.

**Electronic supplementary material:**

The online version of this article (10.1186/s12989-018-0269-x) contains supplementary material, which is available to authorized users.

## Background

The food industry has used titanium dioxide (TiO_2_) since it was approved by the Food and Drug Administration (USA) in 1966 as a food additive [1]. The European Food Safety Authority (EFSA) designated the “E number” E171 to TiO_2_, granting it as a substance that can be used as a food additive [2]. In addition, recent evidence indicates that the use of nanosized titanium dioxide (TiO_2_NPs) in consumer and industrial products has exponentially increased due to their highly valuable refractive, photocatalytic and pigmenting properties [3, 4]. Even though TiO_2_ was classified by the International Agency for Research on Cancer (IARC) as a possible human carcinogen on group 2B in 2010, the Nanotechnology Consumer Products Inventory has documented around 100 consumer products containing TiNPs and TiO_2_NPs to date [5]. Estimations based on the consumption of TiO_2_-containing food lead to the conclusion that, in the US, children and adults may be ingesting around 1–2 and 0.2–0.7 mg/kg bw/day of TiO_2_, respectively [6]. This highlights the relevance of ingestion as an important entryway of TiO_2_ and TiO_2_NPs in human exposures.

Nanotechnology allows the design and synthesis of TiO_2_NPs which present the desired physicochemical characteristics (e.g. shape, phase, and structure) in order to improve, increase, and diversify NPs’ applicability. Therefore, as the range of nanoparticle types and applications increases, the potential toxicities of these novel materials and the properties driving such toxic responses must be fully understood. To date, research with microorganisms has evidenced that the biocidal activity and cytotoxic effects of NPs are structure-, shape-, and size-dependent [7–9]. Furthermore, in vitro mammalian cells’ studies have reported distinct reactive oxygen species (ROS) generation patterns by different-types of TiO_2_ nanowires [10], differences in cytotoxicity between various crystalline-structure TiO_2_NPs [11], and variations in the intracellular accumulation of different crystal-phase food-grade TiO_2_ [12]. It has also been well documented that the crystalline phase and the primary NP diameter alters the biological impact (e.g. biodistribution, toxicokinetics, etc.) of TiO_2_NPs *in vivo* [13–15].

Considering oral exposure as one of the principal entry routes to the human body, the lack of conclusive studies reporting the impact of newly engineered TiO_2_NPs, due to the extreme difficulty of NPs detection, and how they behave across the gastrointestinal tract, is stricking. Accordingly, our study aims to evaluate the biointeractions, biodistribution, and toxicokinetics of TiO_2_NPs in the intestinal barrier, by assessing the biological effects of three differently shaped TiO_2_NPs (nanospheres, nanorods and nanowires). For this purpose, we used an *in vitro* model comprised of Caco-2/HT29 cocultures. After 21 days, the coculture acquires a barrier structure that faithfully mimics the human small intestine epithelium at both the morphological and functional level [16, 17]. Derived from a human colon adenocarcinoma, Caco-2 cells, as enterocyte-like cells, are able to express microvilli, tight junctions (TJ) and present paracellular, transcellular, active and transcytotic transport [18]. Differentiated Caco-2 cells also express all of the major integral membrane enzymes in charge of nutrient hydrolysis, uptake, storage and absorption [19, 20]. In parallel, HT29 cells, known as goblet cells and also derived from a human colon adenocarcinoma, are characterized by their ability to produce and secrete mucus [21].

We have shown that, when seeding at a ratio of 90% Caco-2 to 10% HT29 and culturing for 3 weeks, this in vitro model reaches good integrity levels (> 200 Ω/cm^2^) and is covered by a dense mucus layer, working as a barrier with two distinct scenarios, the lumen and the mucosa [22]. We previously worked in improving a more complex *in vitro* model, the Caco-2/HT29/Raji-B model, which also faithfully reproduces the transcytotic M cells of Payer’s Patches [22]. However, the low amount of M cells along the small intestine, as well the fact that the M-like cells expressed in the *in vitro* model are less than 5% [23], reduces the probability of NPs to be ingested by M-like cells. Consequently, the uptake of NPs by M-cells is difficult to detect by TEM and impossible by confocal microscopy. According to this, we chose the Caco-2/HT29 model to determine whether the mucus layer, as well as the barrier structure, could be compromised by the exposure to TiO_2_NPs. Moreover, we aimed to assess if the potential adverse effects are shape- and structure-dependent by comparing the most commercialized TiO_2_NPs, namely nanospheres (anatase-structure), nanorods (rutile-structure) and nanowires (TiO_2_-structure). For this purpose, we analyzed the barrier’s integrity and permeability after 24 and 48 h of TiO_2_NPs’ exposure, detected cellular uptake and intracellular localization by using laser confocal microscopy, and assessed the barrier functionality by gene expression. In addition, genotoxic and oxidative DNA damage were also evaluated by using the comet assay.

## Methods

### Nanomaterial dispersion and characterization

Three different shapes of titanium dioxide nanoparticles (TiO_2_NPs), pure anatase crystal-structure nanospheres of TiO_2_ (< 25 nm, TiO_2_NPs-S), pure rutile crystal-structure nanorods of TiO_2_ (< 100 nm of diameter, and about 250 nm of length, TiO_2_NPs-R), and nanowires of TiO_2_ (< 10 nm of diameter and 100 μm of length, TiO_2_NPs-W) were purchased from Sigma Chemical Co. (St. Louis, MO). To disperse them, TiO_2_NPs were pre-wetted in 0.5% absolute ethanol and suspended in 0.05% filtered bovine serum albumin (BSA) dissolved in autoclaved MilliQ water. TiO_2_NPs were sonicated in their dispersion medium for 16 min at 10% of amplitude obtaining a dispersed stock of 2.56 mg/mL, according to the Nanogenotox protocol [24]. A complete characterization of the three TiO_2_NPs was carried out to see their behaviour in the cell culture medium used. First, transmission electron microscopy (TEM) was used to determine the dried nanoparticle’s size and morphology on a JEOL JEM-1400 instrument (Jeol LTD, Tokyo, Japan). For this purpose, grids covered with Holey carbon film were immersed carefully in each NPs stock (2.56 mg/mL) and left to dry. Then, TEM images of random fields of view were processed with Image J software to measure and calculate the diameter of 200 NPs. Moreover, the hydrodynamic size and ζ-potential of the three TiO_2_NPs diluted in DMEM cell culture medium (12.5, 50, 100 and 350 μg/mL) were evaluated at 0, 24 and 48 h after sonication by dynamic light scattering (DLS) and laser Doppler velocimetry (LDV) methodologies in a Malvern ZetasizerNano-ZS zen3600 device (Malvern, UK).

### Cell culture and the *in vitro* coculture model

The human colorectal adenocarcinoma cell line Caco-2 was kindly provided by Dr. Isabella Angelis, from *Istituto Superiore di Sanità* (ISS, Italia). HT29, another human cell line derived from a colorectal adenocarcinoma, was purchased from American Type Culture Collection (ATCC, Manassas VA 20108 USA). Both cell lines were maintained in Dulbecco’s modified Eagle’s High Glucose medium without pyruvate (DMEM w/o pyruvate, Life Technologies NY) supplemented with 10% fetal bovine serum (FBS), 1% non-essential amino acids (NEAA) (PAA Laboratories GmbH, Pasching, Austria) and 2.5 mg/mL plasmocin (Invivo Gen, San Diego, CA). Cells were placed in a humidified atmosphere of 5% CO_2_ and 95% air at 37 °C. Routinely, Caco-2 and HT29 cell lines were subcultured once a week with 1% trypsin-EDTA (PAA Laboratories GmbH, Pasching, Austria) at 7.5 × 10^5^ cells/flask and 4 × 10^5^ cells/flask, respectively, in 75 cm^2^ flask.

The *in vitro* coculture model was seeded in 12-well culture plates using a Polyethylene Terephthalate Transwell® (PET) insert with 1 μm pore size and an area of 1.12 cm^2^ (Millipore®) (Merck KGaA, Darmstadt, Germany). Briefly, 1.7 × 10^5^ Caco-2 and HT29 cells clones were mixed and seeded on the apical side of the transwell in a ratio of 90:10, respectively. Finally, Caco-2/HT29 cocultures were left to differentiate for 21 days and the cell culture medium was changed every 3 days.

As indicated in Additional file 1: Figure S1, the Caco-2/HT29 barrier shows a structure similar to the one constituted by Caco-2 monocultures, without relevant overgrown.

### Viability studies

To choose the range of sub-toxic doses to be used in our studies, an initial toxicity study was carried out. Cell viability was determined by the Beckman counter method with a ZTM Series coulter-counter (Beckman Coulter Inc., CA). Twenty-one days-old Caco-2/HT-29 cocultures were exposed for 24 and 48 h to different concentrations of TiO_2_NPs-S, −R and –W, ranging from 0 to 350 μg/mL. After exposure to the given NPs, barriers were washed three times with 0.5 mL of PBS (1%) and incubated 4 min at 37 °C with 0.25 mL of trypsin-EDTA 1%, to detach and individualize the cells. Finally, cells were diluted in ISOTON solution (1/100) and counted with the Beckman Cell Counter. Viability values for each concentration were calculated by averaging three independent viability experiments, each containing three replicates per sample (*n* = 9).

### Evaluation of the barrier’s integrity in the *in vitro* coculture model

To monitor the formation of the differentiated barrier and its integrity, its trans-epithelial electrical resistance (TEER) was measured weekly with a Voltmeter (Millicell-ERS volt/ohm meter). TEER was measured 7, 14 and 21 days after seeding Caco-2/HT29 in PET transwells. Caco-2/HT29 cocultures with TEER values higher than 200 Ω/cm^2^ were used for further experiments. TEER values were also measured after TiO_2_NPs-S, TiO_2_NPs-R, and TiO_2_NPs-W exposure for 24 and 48 h. Briefly, after NPs exposure, the apical and basolateral chambers of the barriers were washed three times with PBS (1%) to remove the NPs as much as possible and fresh DMEM cell culture medium was placed again in the transwells. Each sample was measured three times in different parts of the insert before and after NPs exposure. TEER values for each concentration were calculated by averaging three independent experiments. TEER values were calculated according to the formula TEER = [Ω (cell inserts) - Ω (cell-free inserts)] × 1.12 cm^2^.

### Paracellular transport through the coculture barrier

To support the integrity studies of the Caco-2/HT29 barrier, the paracellular passage of Lucifer yellow (LY) was analyzed. Briefly, after 24 and 48 h of exposure to the different TiO_2_NPs, barriers were washed three times with transport buffer (HBSS; Ca^2+^, Mg^2+^, + 10 mM HEPES, pH 7.4). The inserts were transferred to a new 12-well plate with 1.5 mL of HBSS in the basolateral compartment. LY diluted in HBSS was added to the apical compartment at a final concentration of 0.4 mg/mL and plates were then placed in a 37 °C incubator for 2 h. One hundred μL of each basal compartment was transferred in triplicates to a black 96-well plate. LY leakage through the barrier was measured in a prompt fluorimeter (Victor III, Perkin Elmer) plate reader using a 405–535 nm excitation-emission spectrum.

### TiO_2_NPs localization by confocal microscopy

Laser Confocal Microscopy has demonstrated to be a useful method for localizing metallic NPs inside cells [25]. This method was used to visualize and locate the three different TiO_2_NPs through the cocultured barrier. For this purpose, Caco-2/HT29 barriers were exposed to 150 μg/mL of TiO_2_NPs-S, −R and -W for 24 and 48 h. After the exposure time, barriers were stained in situ with *Hoechst 33,351* and *WGA Alexa Fluor™*, diluted in DMEM cell culture medium at concentrations of 1/500 and 1/100, respectively, for 15 min. Images were obtained by using a confocal laser scanning microscope Leica TC2 SP5. The three types of TiO_2_NPs were visualized thanks to their own reflective capability and manually masked with green colour, in contrast with the blue colour of the cells’ nucleus and the red colour or the extracellular membrane and the mucus layer. Confocal images were processed with the software Huygens Essential 4.4.0p6 (Scientific Volume Imaging, Netherlands), and Imaris 7.2.1 (Bitplane, AG).

### TiO_2_NPs transport across the Caco-2/HT29 coculture barrier

To detect the TiO_2_NPs crossing through the Caco-2/HT29 barriers, laser confocal microscopy was also used. To discern if TiO_2_NPs’ transport was shape-, concentration- or time-dependent, coculture barriers were exposed to different concentrations (12.5, 50, 100, and 350 μg/mL) of TiO_2_NPs-S, −R and -W for 24 and 48 h. After the NPs’ exposure, the cell culture medium (1.5 mL) in the basolateral compartment was collected. To eliminate the inorganic material aggregates and crystallized proteins, samples were treated with proteinase K (100 μg/mL) during 30 min at 37 °C. Next, samples were centrifuged in a speed vacuum at 37 °C for 2 h to concentrate the NPs present in the medium. Finally, 10 μL of each sample was placed in slides, covered with a cover-slip, and the NPs were observed under the confocal microscopy. Several images were taken from random fields of each sample. Confocal images were processed with Huygens Essential 4.4.0p6 (Scientific Volume Imaging, Netherlands) and Imaris 7.2.1 (Bitplane, AG) softwares, where the percentage of the reflective area of each sample was calculated. Semi-quantitative values were obtained from three different experiments.

### RNA extraction and gene expression by real-time qPCR

Total RNA from Caco-2/HT29 coculture barriers exposed to 0, 50 and 150 μg/mL of TiO_2_NPs-S, −R and –W, for 24 and 48 h, was extracted using TRIzol® Reagent (Invitrogen, USA) following the manufacturer’s instructions. RNase-free DNase I (DNA-free TM kit; Ambion, UK) was used to discard residual DNA contamination. The first-strand cDNA synthesis kit (Roche, Basel, Switzerland) was used to obtain cDNA from 100 ng of total RNA. The resulting cDNA was subjected to real-time PCR analysis on a LightCycler-480 to evaluate the relative expression of the brush border enzymes sucrase-isomaltase, alkaline phosphatase, and solute carrier family. Gene expression of tight junction components such as *claudin 2*, *zonula occludens*, and *occludin*. The expression of *β-actin* was used as the housekeeping control. The primer sequences are summarized as a table in Additional file 1: (Table S1). Each 20 μL of reaction volume contained 5 μL cDNA, 10 μL of 2× LightCycler 480 SYBR Green I Mater (Roche, Germany), 3 μL of distilled H_2_O and 1 μL of each primer pairs at a final concentration of 10 μM. The cycling parameters were the following: an initial step of 95 °C for 5 min, then 45 cycles of 95 °C for 10 s, 62 °C for 15 s and 72 °C for 25 s. Cycle time (Ct) values were calculated with the LightCycler 480 software package and then normalized with β-Actin Ct values.

### Genotoxic and oxidative DNA damage quantification

The potential induction of genotoxic and oxidative DNA damage in Caco-2/HT29 coculture barriers was assessed by the alkaline comet assay after 24 and 48 h of exposure to TiO_2_NPs-S, −R, and W treatments. The concentration-range was 0, 12.5, 50, 150, and 350 μg/mL for all the TiO_2_-shapes. The addition of formamidopyrimidine-DNA glycosylase (FPG enzyme) was used to measure oxidatively-damaged DNA bases. The used FPG was a gift from Prof. Andrew Collins (University of Oslo). Briefly, once treated, barriers were washed twice with PBS, trypsinized (1% trypsin), and centrifuged at 1000 rpm for 8 min. The pellet was then resuspended in PBS to a concentration of 700 cells/μL and placed in ice at 4 °C, to avoid DNA repair. 25 μL of cells’ suspension was mixed with 0.75% of LMP agarose at 37 °C and dropped (7 μL/drop and 3 drops/sample) on Gelbond (GB) films. Cells on GB were lysed in lysis buffer at 4 °C and pH 10 overnight. The next morning, GB were washed twice (1 × 5 min, and 1 × 50 min) in enzyme buffer at 4 °C and pH 8.0, followed by a 30 min incubation with the enzyme buffer at 37 °C. One GB was incubated with enzyme buffer and FPG enzyme (1/10.000), and the other in enzyme buffer without FPG. GB were incubated with electrophoresis buffer (alkaline buffer) for 35 min followed by the electrophoresis step for 20 min at 20 V and 300 mA at 4 °C. Finally, GB were rinsed twice in cold PBS for 5 min, in distilled water for 1 min, fixed in absolute ethanol for at least 2 h, air-dried overnight at room temperature, and stained with SYBR Gold for 20 min. Each GB film was cut into two similar-sized parts to fit in an acrylic slide (52.5 × 75 × 3 mm). A coverslip of 52.5 × 75 mm was placed on top of the drops, effectively sealing the samples. GB were observed using an epifluorescent Olympus BX50 and damage was quantified measuring the percentage of DNA in tail by using the Komet 5.5 Image analysis software. One hundred randomly-selected comet images were analyzed per sample. 30 min treatments of 5 mM of potassium bromate (KBrO_3_) and 2.5 mM of methylmethanesulfonate (MMS) were used as positive control of oxidative and genotoxic damage, respectively.

### Statistical analysis

All measurements were made in triplicates, at least for 2 separate experiments. Results are expressed as mean ± standard error. One-way ANOVA with Tukey’s post-test, unpaired and paired Student’s *t*-test or two-way ANOVA were used to compare differences between means. Data were analyzed with GraphPad Prism version 5.00 for Windows (GraphPad Software, San Diego California USA, http://www.graphpad.com). Differences between means were considered significant at *P* < 0.05.

## Results

### Nanoparticles characterization

**Fig. 1:**
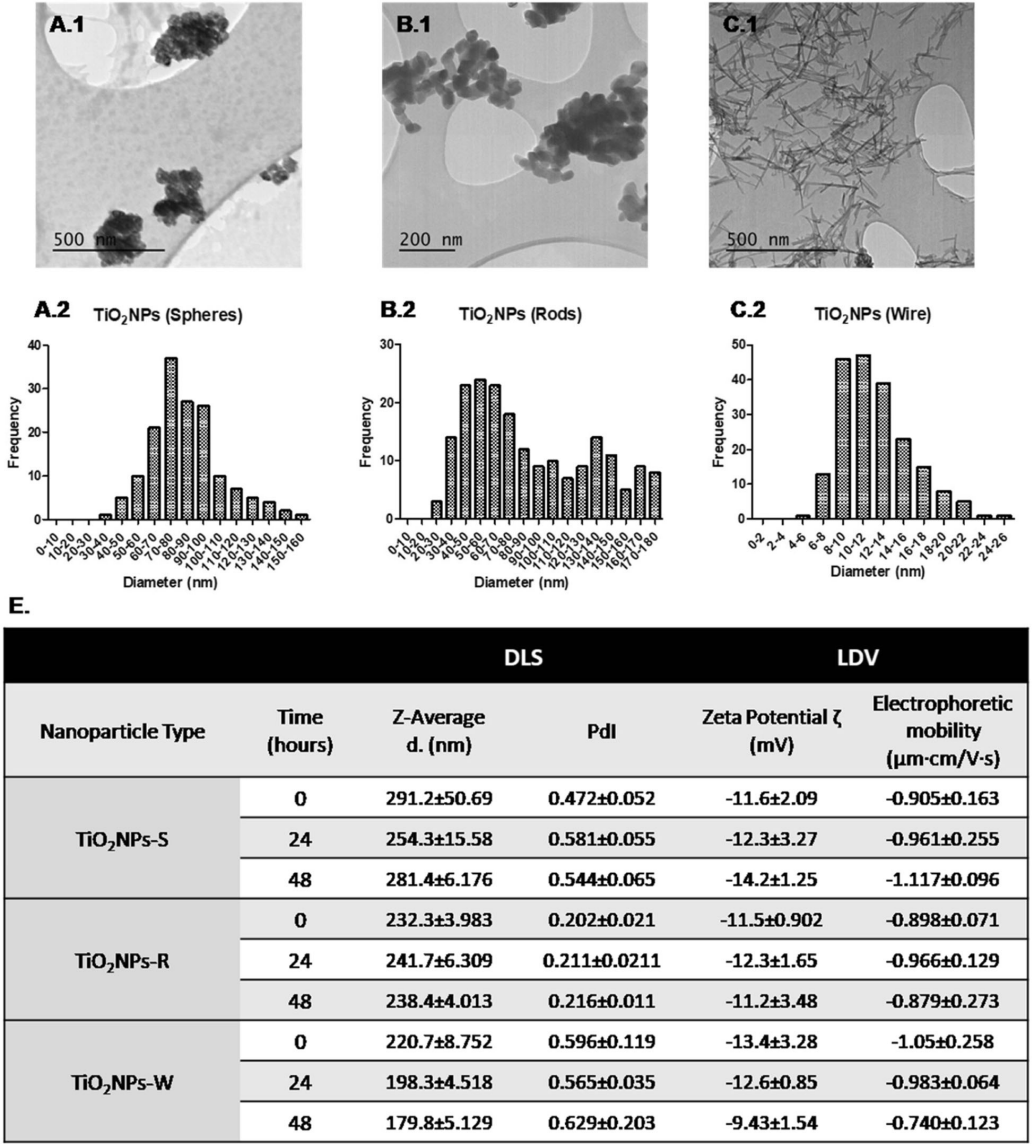
TiO_2_NPs characterization. TEM images of TiO_2_NPs-S (*A.1*), TiO_2_NPs-R (*B.1*), and TiO_2_NPs-W (*C.1*) in their dry form. Size distribution of TiO_2_NPs-S (*A.2*), TiO_2_NPs-R (*B.2*), and TiO_2_NPs-W (*C.2*) of 200 randomly-selected nanoparticles. (*E*) Dynamic light scattering (DLS) and laser Doppler velocimetry (LDV) measurements of 50 μg/mL TiO_2_NPs over the exposure time (0, 24, and 48 h). Data are represented as mean ± SD

Our TEM images demonstrate that the sizes of TiO_2_NPs-S, TiO_2_NPs-R and TiO_2_NPs-W ranged from 70 to 80, 40–70 and 8–14 nm, respectively (Fig. 1; A.1, A.2 and A.3), which are similar to the sizes given by the manufacturer. In spite of the dry form sizes of the NPs, the hydrodynamic diameter measured with the DLS technique gives higher values for the three TiO_2_NPs, reaching mean diameters above 200 nm in most cases (Fig. 1e). These differences between primary and hydrodynamic size suggest that TiO_2_NPs aggregate in the cell culture medium. No significant changes were seen in size distribution for -S or -R forms in cell culture medium (DMEM) over the incubation time. However, a slight size reduction in -W was observed after 48 h. As differences between TiO_2_NPs structures and shapes were detected, we also aimed to study the hydrodynamic size distribution according to the used concentrations (Additional file 1: Figure S2). Interestingly, different behaviours among TiO_2_NPs were observed: -S presented a tendency to aggregate that was clearly concentration-dependent, as higher concentrations correlated with bigger NPs. On the contrary, no concentration nor time-dependent correlations were observed for TiO_2_NPs-R. Regarding -W size distribution, only the lowest concentration (12.5 μg/mL) decreased its aggregation over time, while the higher concentrations presented similar sizes distributions. In spite of the inaccuracy of the size data for nano-filaments measurements, DLS gives important information about the agglomeration state of our dispersion. As nanowires agglomerate, then move slower in the dispersion medium and the average diameter value is bigger [26]. PDI values were higher in -S (0.472, 0.581 and 0.544) and in -W (0.596, 0.565 and 0.629) than in -R (0.202, 0.211 and 0.216), suggesting that -R have greater monodisperse size distribution than the others.

No changes in PDI values were detected over time for any of the evaluated TiO_2_NPs. The stability of the colloidal system was measured by LDV, indicating the TiO_2_NPs surface charge when dispersed in cell culture medium. Our results evince little stability in all TiO_2_NPs solutions since the ζ-potential values barely reach the ±30 mV (Fig. 1e).

### Cytotoxic effects of the Caco-2/HT29 coculture barrier exposed to TiO_2_NPs

**Fig. 2:**
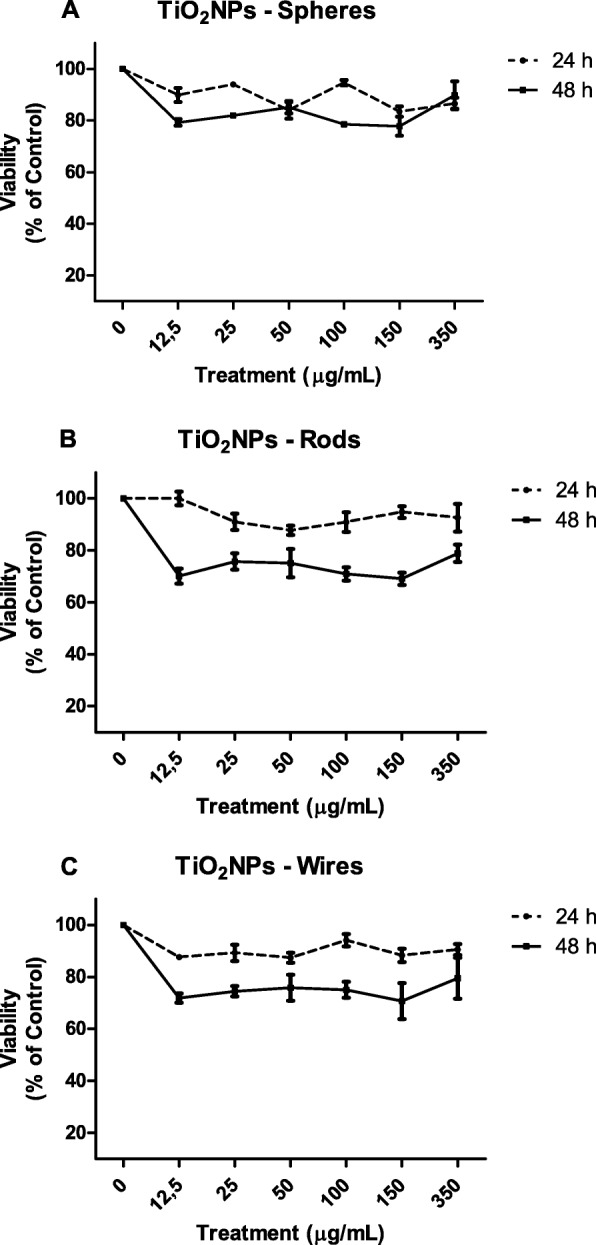
Cell viability (% of control) of Caco-2/HT29 co-culture barrier treated with 0–350 μg/mL of TiO_2_NPs-S (**a**), TiO_2_NPs-R (**b**), and TiO_2_NPs-W (**c**) for 24 or 48 h. Results were analyzed according to the one-way ANOVA with Tukey’s post-test and are represented as mean ± SEM

To determine the cytotoxic effects of TiO_2_NPs-S, −R and –W, and to know whether their shape and titanium-based structure play a role in cell viability, we exposed the 21-days cocultures to concentrations ranging from 12.5 to 350 μg/mL for 24 and 48 h. In Additional file 1: Table S3, we indicated the relationship between μg/mL and μg/cm^2^. Since ingested TiO_2_NPs have low absorption in rats [27], and human ingestion has shown to be daily [6], we evaluated the effects at two different times (24 and 48 h). As Fig. 2 indicates, noncytotoxic damage was detected after 24 h of -S, −R, and -W exposures, as all the registered viability values were above the 80%. Nevertheless, when cell viability was checked after 48 h of exposure, a drastic decrease in cell viability was observed for the three TiO_2_NPs-shapes, although these effects were not concentration-dependent. Interestingly, the concentration of 150 μg/mL seems to be the most toxic since it caused the highest mortality in all the TiO_2_NPs tested at 48 h. In spite of the observed toxicities, we can conclude that shape can be associated with adverse effects as cytotoxicity.

### Evaluation of the barrier’s integrity after TiO2NPs’ exposure

**Fig. 3:**
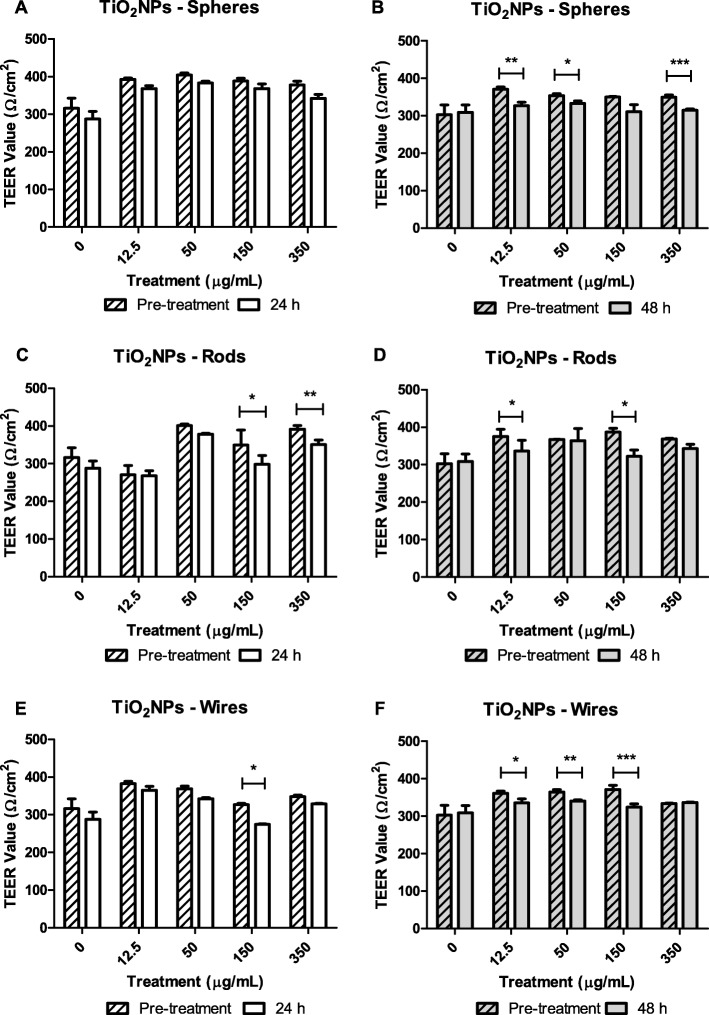
TEER measurements of Caco-2/HT29 co-culture barriers before and after 24 and 48 h of exposure to TiO_2_NPs-S (**a** and **b**), TiO_2_NPs-R (**c** and **d**), and TiO_2_NPs-W (**e** and **f**). Results were analyzed with a paired Student’s *t-*test and represented as mean ± SEM. *P < 0.05, **P < 0.01, ***P < 0.001

The main function of an epithelial barrier is to confer stability, protection, and the desired permeability to each tissue and/or organ. In these functions, TJ play an important role forming belt-like and apical-most adhesive junctional complexes around mammalian cells [28]. We can evaluate the functional integrity of the barrier measuring the TEER before and after the exposure to TiO_2_NPs. No significant reduction was seen in the integrity of the barrier when exposed to TiO_2_NPs-S for 24 h (Fig. 3a). However, significant differences (*P* < 0.05) between TEER values were observed by exposing the barriers to 150 μg/mL of both TiO_2_NPs-W (Fig. 3c), and TiO_2_NPs-R (Fig. 3e). Moreover, rod-shaped NPs were also able to decrease the membrane’s stability at 350 μg/mL (*P* < 0.001). When the exposure time was extended to 48 h, statistically significant adverse effects on the barrier’s integrity were detected for all TiO_2_NPs shapes at different NPs concentrations (Fig. 3b, d and f), although no concentration-dependent effect was observed.

**Fig. 4:**
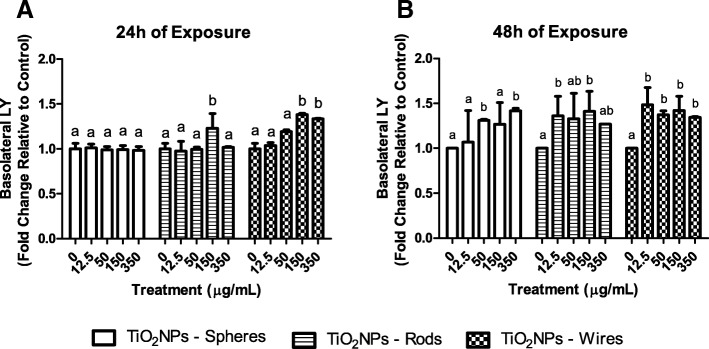
Percentage of LY found in the basolateral chamber of the transwell. The paracellular transport of LY was measured after treating the Caco-2/HT29 co-culture barriers with TiO_2_NPs-S, TiO_2_NPs-R, and TiO_2_NPs-W for 24 (**a**) or 48 h (**b**). Results represented as mean ± SEM. Bars that do not share any letter are significantly different according to the one-way ANOVA with Tukey’s post-test (*P* < 0.05)

A reduction in the barrier’s integrity and stability may cause increased permeability to a wide range of endogenous and exogenous particles and/or substances. Thus, we initially focused our integrity studies on analyzing the possible variations of paracellular transport, measuring the pass of LY across the barrier after TiO_2_NPs exposure. The results obtained agree with the TEER variations observed previously. As we can see in Fig. 4, no increases in the ratio of basolateral LY were observed when the barrier was exposed for 24 h to TiO_2_NPs-S. However, the highest concentrations of TiO_2_NPs-R, and –W significantly incremented LY’s passage (Fig. 4a). Also, 48 h exposures to all the three TiO_2_NPs induced significant increases in basolateral LY concentrations when compared to the control. Summarizing, our results show that all TiO_2_NPs disrupt the cell membrane’s integrity and permeability by increasing its paracellular transport. Interestingly, exposure to TiO_2_NPs-W was the most harmful, modifying stability parameters in most of the experimental conditions.

### Assessing detrimental effects of TiO_2_NPs exposure by gene expression

To support our integrity and permeability results, and to evaluate the barrier status, changes in gene expression of several markers associated with different intestinal barrier functions were analysed. To this aim, the Caco-2/HT29 coculture was exposed to 50 and 150 μg/mL of TiO_2_NPs-S, −R, and –W, for 24 and 48 h. We analyzed changes in the expression of genes related to nutrient uptake and digestion, as well as in genes responsible for sealing intercellular spaces, thus conferring the barrier function. This set of genes, their encoded proteins and their functions are summarized in Table 1.

**Table 1:** Genes encoding molecular markers of the Caco-2/HT29 barrier, and analysed by RT-qPCR

Gene Identification	Encoded protein name	Function
*ALPI*	Intestinal alkaline phosphatase	Digestive brush-border enzyme. Detoxification of lipopolysaccharides
*SI*	Sucrase-isomaltase	Digestion of dietary carbohydrates including starch, sucrase and isomaltase
*SLC15A1*	Solute carrier family 15 member 1	Intestinal hydrogen peptide cotransporter. Uptake of di- and tri-peptides from the lumen and into enterocytes
*ZO-1*	Zonula occludens-1	Tight junction adaptor protein that also regulates adherent junctions.
*OCLN*	Occludin	Integral membrane protein. Required for cytokine-induced regulation of the tight junction paracellular permeability barrier.
*CLDN2*	Claudin-2	Claudin proteins are identified as major integral membrane proteins, localized exclusively at tight junctions in the intestine.

**Fig. 5:**
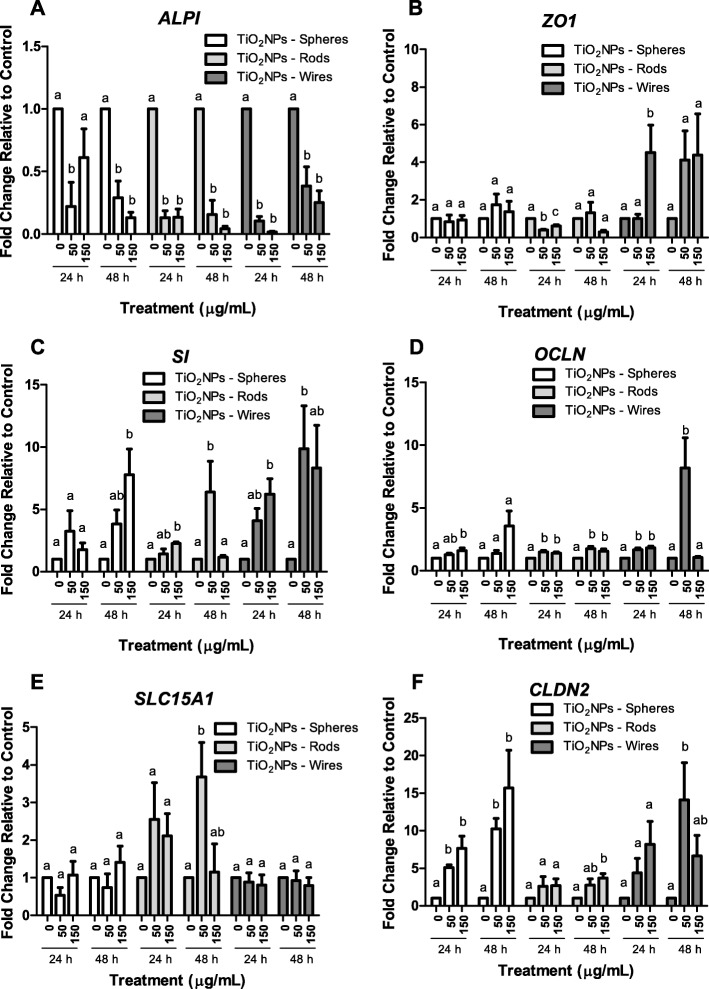
Gene expression of Caco-2/HT29 molecular markers in response to 24 or 48 h of TiO_2_NPs-S, TiO_2_NPs-R, and TiO_2_NPs-W exposure. Results represented as mean ± SEM. Bars that do not share any letter are significantly different according to the one-way ANOVA with Tukey’s post-test (*P* < 0.05)

Interestingly, we observed a significant and consistent down-regulation of *ALPI* in all exposure conditions and for all the TiO_2_NPs shapes tested (Fig. 5a). Contrarily, significant increases in *SI* expression were detected after 24 and 48 h of TiO_2_NPs-R, and -W exposure, while -S exposure was able to upregulate this gene’s expression only after 48 h (Fig. 5c). *SLC15A1* gene expression also increased significantly, but only when the barrier was exposed to 50 μg/mL TiO_2_NPs-R for 48 h (Fig. 5e). Summarizing, the expression of different enzymatic functions could be affected distinctly depending on the TiO_2_NPs structure, dose and time, either by enhancement or by reduction.

Regarding the gene expression changes of the main integral membrane proteins located at the TJ (*OCLN*, *CLDN2* and *ZO-1*), results were more homogeneous. Generally, the exposure to TiO_2_NPs-S did not significantly modify the gene expression of *ZO1* at any time nor concentration. Conversely, *ZO1* was significantly downregulated when the barrier was exposed to 50 and 150 μg/mL of TiO_2_NPs-R for 24 h, while 150 μg/mL TiO_2_NPs-W exposure upregulated *ZO1* expression after 24 h (Fig. 5b). *OCLN* was upregulated after 24 h of exposure to TiO_2_NPs-S and also to TiO_2_NPs-R, and –W, both after 24 and 48 h (Fig. 5d). Finally, *CLDN2* was significantly upregulated in all experimental conditions after exposing the barrier to TiO_2_NPs-S. However, only exposure to TiO_2_NPs-R and -W for 48 h upregulated *CLDN2* expression (Fig. 5f). Taken together, these data suggest that the NPs’ shape could interact distinctly with the junctional complex modulating different responses.

### Caco-2/HT29 barrier uptake of TiO_2_NPs

**Fig. 6:**
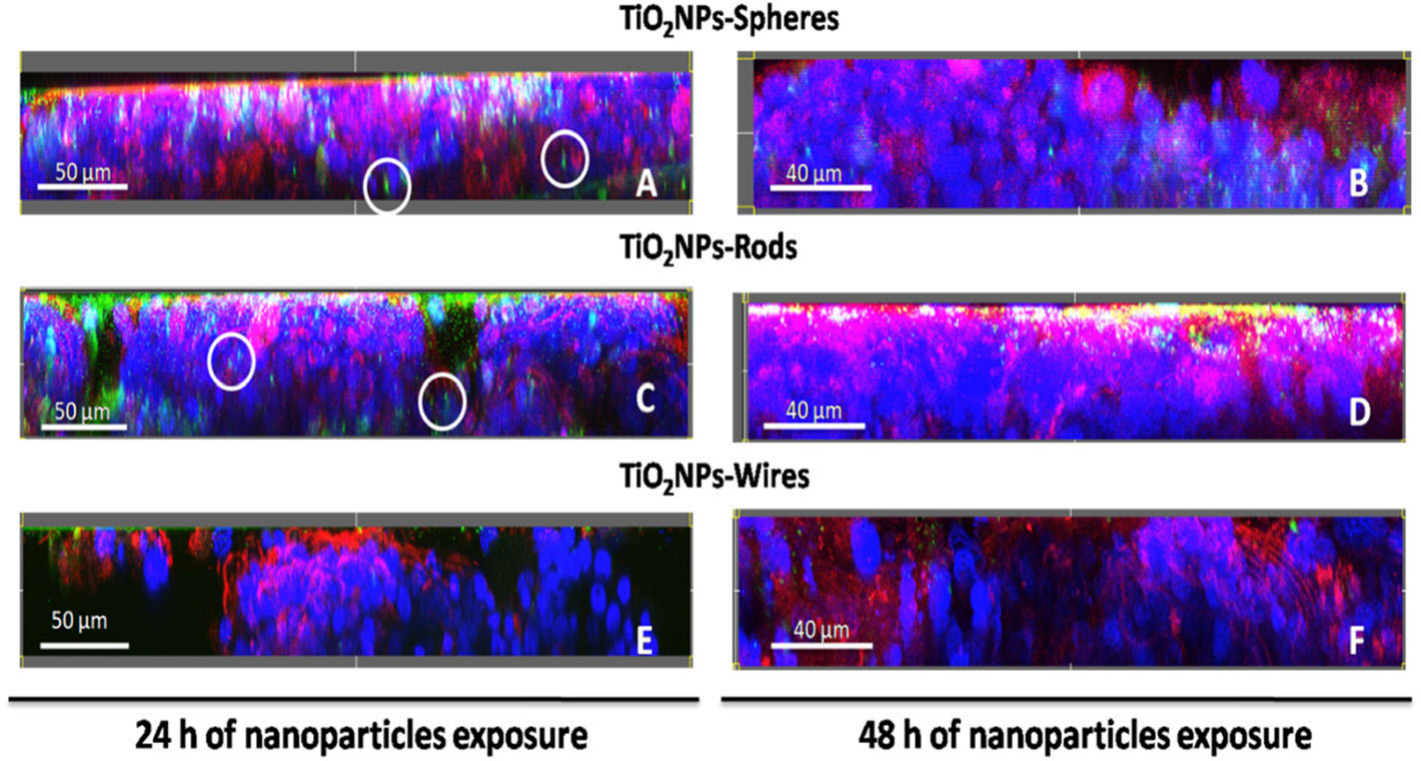
Confocal microscope z-scans of Caco-2/HT29 co-culture barriers after TiO_2_NPs-S (**a** and **b**), TiO_2_NPs-R (**c** and **d**), and TiO_2_NPs-W (**e** and **f**) exposures lasting for 24 h (**a**, **c** and **e**), or 48 h (**b**, **d** and **f**). Cell nuclei (blue) were stained with Hoechst and cell membrane and mucus (red) with WGA. NPs were visualized by reflection and marked with a green mask. Images were processed with the Imaris 7.2.1 software

Confocal microscopy was used to qualitatively localize TiO_2_NPs in our barrier model, specifically in each of its components (i.e. mucus shed, cell cytoplasm, cell nuclei, apical and basal areas, etc.). As metallic NPs have the capability to reflect polarized light, their detection results easier by confocal microscope than by electron microscopy. Furthermore, we aimed to study whether the NPs’ structure and shape could influence uptake and/or translocation, and to check potential biointeractions and biodynamics over the exposure time. Briefly, the status of Caco-2/HT29 coculture barriers was analyzed after 24 h and 48 h exposures to 150 μg/mL of TiO_2_NPs-S, TiO_2_NPs-R, and TiO_2_NPs-W. Figure 6 shows confocal images corresponding to transversal cuts of the barrier, where it is possible to distinguish the cell nucleus in blue, the TiO_2_NPs in green, and the mucus secretions and cell membranes in red. As observed, after 24 h of exposure most of the TiO_2_NPs-S and TiO_2_NPs-R remained sedimented and/or attached to the apical side of the barrier, where the microvilli and mucus shed form an extracellular environment suitable for the NPs immobilization (Fig. 6a and c). Although not in a quantitative manner, we can assume that the trapped NPs in the apical side are more aggregated/agglomerated than those located deeper in the barrier (white circles). Interestingly, the amount of TiO_2_NPs-S detected in the apical part of the barrier was clearly reduced after 48 h of NPs exposure, while the amount of TiO_2_NPs-R was similar at both time points. As images E and F from Fig. 6 show, after 24 and 48 h of exposures to 150 μg/mL of TiO_2_NPs-W the barrier looked more damaged and compromised at a structural level when compared to the other TiO_2_NPs exposures. Also, the amount of internalized TiO_2_NPs-W, at both time points, was markedly lower.

**Fig. 7:**
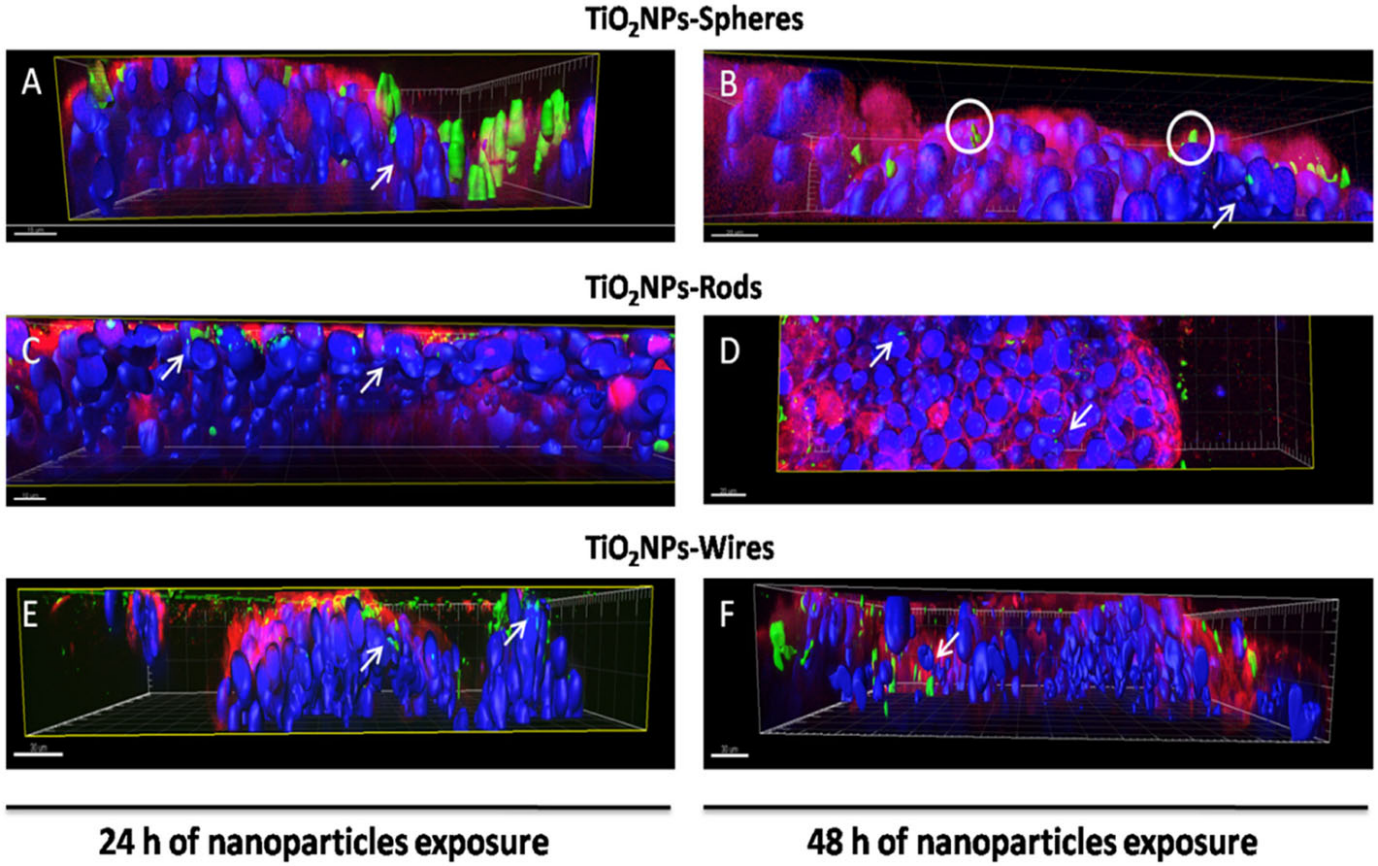
Three-dimensional confocal images of the Caco-2/HT29 co-culture barriers *z*-scans from Fig. 6. Images were taken after exposures of 24 or 48 h to TiO_2_NPs-S (**a** and **b**), TiO_2_NPs-R (**c** and **d**), and TiO_2_NPs-W (**e** and **f**). Cell nuclei (blue) were stained with Hoechst, and mucus (red) was stained with WGA. NPs were visualized by reflection and marked with a green mask. White arrows indicate NPs in the cell cytoplasm and NPs-nucleus interactions. Images were processed with the Imaris 7.2.1 software

Using the confocal technique, we were able to elucidate the exact location of TiO_2_NPs in the cocultured cells, although the identification of each cell type was not possible. As white arrows indicate (Fig. 7), TiO_2_NPs-S (A), −R (C), and -W (E) were detected in the cell cytoplasm after 24 h of exposure, and in most cases, they reached the cell nucleus. When evaluating the three-dimensional images from samples exposed to the three different TiO_2_NPs shapes for 48 h, lower amounts of TiO_2_NPs-S were still immobilized in the apical part of the membrane (white circle), and fewer NPs were detected inside the cells (white arrows) (Fig. 7b). Similar results were obtained when the barriers were exposed to TiO_2_NPs-R (Fig. 7d), and TiO_2_NPs-W (Additional file 1: Figure S3) for 48 h. As previously observed, fewer cell junctions and cohesion were found when checking in detail the cocultures exposed to TiO_2_NPs-W for both 24 (Fig. 7e) and 48 h (Fig. 7f). Moreover, TiO_2_NPs-W were clearly detected at different levels of the barrier width (white arrow).

### Nanoparticles translocation through the Caco-2/HT29 barrier

**Fig. 8:**
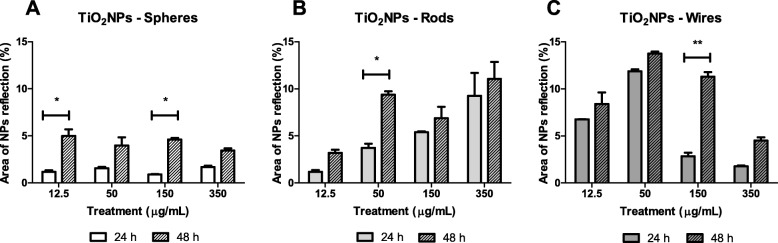
Percentage of the TiO_2_NPs-reflective area of the basolateral chamber of the transwell. The transport of TiO_2_NPs-S (**a**), TiO_2_NPs-R (**b**), and TiO_2_NPs-W (**c**) across the Caco-2/HT29 co-culture barrier was calculated using Laser Confocal microscopy and by measuring the reflected light of randomly selected slide fields. Data are represented as mean ± SEM

As the capability of the three TiO_2_NPs to penetrate the Caco-2/HT29 coculture barrier was detected, we aimed to find and quantify any amount of translocated TiO_2_NPs by analyzing the basolateral medium of the barrier model. Our previous experience working with Caco-2 monolayers demonstrates that confocal microscopy is one of the best techniques to localize metallic NPs in the basolateral media. Thereby, after 24 or 48 h of TiO_2_NPs-S, −R, and -W exposure, we collected the entire basolateral medium (1.5 mL), treated it with proteinase *K,* and concentrated the sample by evaporating the cell medium using a speed vacuum. After that, each sample was analyzed under the confocal microscope, where several pictures were taken in random fields of a slide (Additional file 1: Figure S4). Semi-quantitative data was obtained by measuring the percentage of the area reflected by the different TiO_2_NPs. As Fig. 8 shows, the transport of TiO_2_NPs-S through the barrier was not concentration-dependent. However, the amount of NPs found in the basolateral chamber 48 h after exposure (~ 5% of the area) was almost two times higher than at 24 h (~ 2% of the area). On the other hand, the TiO_2_NPs-R transport was clearly concentration- and time-dependent. Finally, although TiO_2_NP-W in the basal growth medium also increased with the exposure time, nanowires behave differently than the rods, as its transport decreased when the exposure concentration increased.

### Genotoxic and oxidative damage. The comet assay

**Fig. 9:**
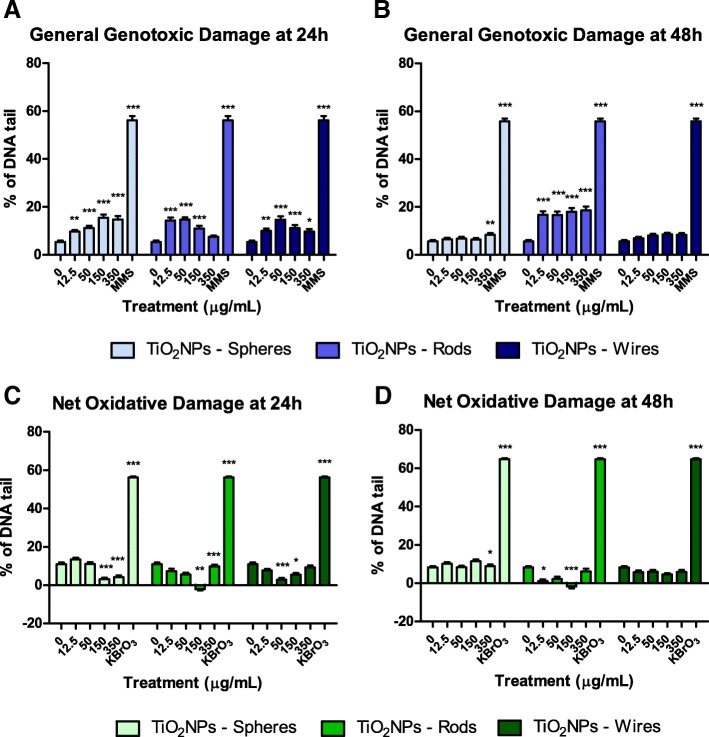
DNA damage studies using the Comet assay complemented with the FPG enzyme. Genotoxic damage observed after 24 (**a**) or 48 (**b**) h of exposure to TiO_2_NPs (-S, −R and -W). Mean oxidative damage observed after 24 (**c**), and 48 (**d**) h of exposure to TiO_2_NPs (-S, −R and -W). (*) denotes significant differences according to the one-way ANOVA with Tukey’s post-test (*P < 0.05, **P < 0.01***P < 0*.*001). Results are represented as mean ± SEM

The comet assay was used to analyze the consequences of the TiO_2_NPs-cell nucleus interaction previously observed by confocal microscopy. Moreover, we also aimed to elucidate if this response was concentration and/or time-dependent. As depicted in Figs. 9, 24 h of TiO_2_NPs-S, TiO_2_NPs-R, and TiO_2_NPs-W exposure significantly increased the general genotoxic damage in our barrier model (Fig. 9a). Nevertheless, after 48 h of TiO_2_NPs exposure, only those barriers exposed to TiO_2_NPs-R sustained a non-concentration dependent genotoxic damage (Fig. 9b). Methyl methanesulphonate (MMS), a well-known genotoxic compound used as positive control, clearly induced general genotoxic damage to the cocultured cells (Fig. 8a and b).

The potential ability to induce oxidative damage was also detected performing the alkaline version of the comet assay, where oxidatively-damaged DNA bases (e.g. 8-oxodG and FAPydG) were detected using the formamidopyrimidine glycosylase enzyme (FPG), thereby increasing the number of DNA breaks. The difference in the percentage of DNA in tail between cells treated with FPG and those left untreated gave us a measure of the amount of oxidative DNA damage [29, 30]. In this case, no significant increase in oxidative DNA damage was detected after 24 h of TiO_2_NPs-S, −R and -W exposure, or after exposures lasting for 48 h. As a positive control, we used the well-known oxidant agent, potassium bromate (KBrO_3_), which increased a 60% the oxidative DNA damage at the Caco-2/HT29 barrier.

## Discussion

The International Agency for Research on Cancer (IARC) performed the last reevaluation on the potential cancer risk of TiO_2_ in 2010 [31]. According to the collected information, TiO_2_ was classified as a potential human carcinogen in group 2B, because there was enough evidence that inhalation of nano-TiO_2_ may cause lung cancer. Although IARC considered the risk associated with oral exposure, inconclusive outcomes were obtained due to the lack of standardized procedures for nano-TiO_2_ risk assessment, as pointed out by Jacobs et al. [32]. Under this framework, we aimed to investigate the potential hazard of three different shapes and crystal structures of TiO_2_NPs using an *in vitro* model of intestinal barrier constituted by Caco-2 and HT29 cells cocultures.

A preliminary characterization of the selected TiO_2_NPs showed larger hydrodynamic diameter values than their primary sizes. This would agree with other studies showing the general tendency of NPs, and of TiO_2_NPs in particular, to form agglomerates in cell culture media [33, 34]. Interestingly, our results showed that crystallinity and morphology are not influential factors in determining the stability of TiO_2_NPs suspensions, which agrees with previously reported data [35]. Although most of the studies barely take into account the potential role of incubation time when characterizing NPs, we strongly believe that this should be a prerequisite to understanding the effects of the exposure time and their multiple potential biological effects. Accordingly, we took this parameter into consideration but we did not detect relevant variations in the hydrodynamic size for TiO_2_NPs-S or TiO_2_NPs-R over the incubation time (0, 24, and 48 h). Nevertheless, a slight reduction in the hydrodynamic size for TiO_2_NPs-W was observed, which would indicate the lack of time-dependent aggregation. The agglomeration status of NPs can influence the potential toxicity by changing their uptake and/or the way they interact with cells. In fact, changes in this parameter are considered as one of the main reasons for the contrasting data reported in different studies [33, 36].

Even though exposure to TiO_2_NPs occurs chronically in humans, it should be pointed out that cells from the gastrointestinal tract are submitted to a high turnover. Contrarily to this chronic in vivo exposures, most of the *in vitro* studies testing potential biological effects are performed evaluating effects over a short exposure time (e.g. after 4, 6 or 24 h). These short exposure times do not reflect the observed *in vivo* NPs effects [37]. To increase the fidelity of *in vitro* models, elongating the exposure time and minimizing the exposure concentration could be a useful approach; however, the increase of the exposure times (6, 24 and 48 h) did not cause variations in the observed effects on Caco-2 monolayers [38]. Going one step further, we have been able to detect a significant reduction in cell viability after 48 h for all TiO_2_NPs-shaped exposures using our Caco-2/HT29 coculture model, which points out the relevance of exposure time. Nonetheless, our results are in contradiction with those observed in Caco-2/HT29-MTX cocultures, where no cytotoxic effects were observed after similar exposure conditions [38, 39]. As previously stated, the agglomeration status after 48 h of TiO_2_NPs suspended in serum-containing cell culture medium was smaller for TiO_2_NPs-R and TiO_2_NPs-W and similar for TiO_2_NPs-S in our case. This could be a potential factor explaining the increased cytotoxicity over the exposure times. In addition, our model, using the HT29 clone, and the proportion 90% of Caco-2 and 10% of HT29, has demonstrated a proper mucus secretion that spreads all over the surface of the barrier, forming a thick mucus layer and conferring a good protection and integrity [22]. Since Dorier et al. [38] and Brun et al. [39] used a different HT29 cell clone, and their cocultures were composed by 70% of Caco-2 and 30% HT29-MTX, these factors would explain the observed differences and, at the same time, would reinforce the usefulness of our model.

To characterize the effects of the interaction of the three TiO_2_NPs shapes with the barrier, we assessed its functionality and permeability by TEER and LY paracellular crossing. Although TiO_2_NPs-S did not disrupt the integrity of our model after 24 h exposures, small but significant effects were observed for TiO_2_NPs-R and –W at this time point, pointing out the relevance of the NPs’ shape when analyzing its effects. The extension of the exposure time enhanced the damaging effects observed at 24 h, as the three different shapes of TiO_2_NPs affect the barrier’s integrity after exposures lasting for 48 h. Interestingly, the observed adverse effects on the barrier’s integrity depend on the shape of the TiO_2_NPs used as well: TiO_2_NPs-W causes the most adverse effects, while TiO_2_NPs-S is the least hazardous. Moreover, the observed upregulation in genes encoding different TJ components (*ZO*-1, *OCLN,* and *CLDN2*) indicates the presence of active repair by inducing de novo expression of junctional proteins [39], confirming the cell junctions’ and the barrier’s integrity impairment.

After NPs ingestion, they can interact with a number of biologically significant tissues and structures, as the enterocytes’ brush border (microvilli). In fact, it has already been observed that the adsorption of NPs results in the disruption of the brush border’s structure [40, 41], where approximately 42% of microvilli were lost after the exposure to food-grade TiO_2_ [42]. In our case, we have found that exposures to different TiO_2_NPs’ shapes also altered significantly the Caco-2/HT29 brush border membrane and enzymatic function, as the expression of *ALPI* was readily and radically downregulated after exposure to all of the TiO_2_NPs. It must be noted that a recent study analyzing ALPI’s enzymatic activity in Caco-2/HT29-MTX cocultures observed an increased activity after acute and chronic TiO_2_NPs exposures [43]. These discrepancies between post-transcriptional and post-translational regulations lead us to hypothesize that an enzyme over-activation of ALPI in response to TiO_2_NPs might trigger the block of its gene transcription to mRNA. In addition, the *SI* gene expression was upregulated after most of the exposure conditions analyzed, suggesting an active response to carbohydrates starvation caused by the TiO_2_NPs exposure. Finally, the upregulation of *SLC15A1* gene expression was detected after TiO_2_NPs-R exposure, but not after TiO_2_NPs-S and TiO_2_NPs-W ones. This would agree with what was observed in Caco-2 monolayers exposed to rutile TiO_2_NPs for 48 h [12]. Overall, TiO_2_NPs exposure affects the barrier’s integrity, but each brush border enzyme may follow different strategies to stabilize its correct function, independently of the concentration exposure and NP shape.

The disruption of the barrier integrity can potentially affect cellular uptake and translocation throughout the barrier of the TiO_2_NPs, making the evaluation of these parameters necessary to evaluate the risk of exposure. A wide number of mammalian cell type models internalize TiO_2_NPs, including differentiated Caco-2 cells [41, 44]. Interestingly, the capability of differentiated Caco-2 cells to internalize TiO_2_NPs depends on their structure, as more rutil- than anatase-structured TiO_2_NPs were observed in differentiated Caco-2 [12, 39]. When the Caco-2/HT29 cocultures are established, technical difficulties to follow and locate the NPs position inside individualized Caco-2 or HT29 cells appear. To elucidate the TiO_2_NPs distribution in our *in vitro* model of the intestinal barrier, we used laser scanning confocal microscopy. With this methodological approach, we detected that (i) numerous TiO_2_NPs-S and -R agglomerates remain deposited and entrapped in the apical part of the barrier, where the mucus shed and microvilli are located; while the smaller agglomerates could penetrate the barrier deeper; (ii) TiO_2_NPs-R were more confined between mucus and microvilli than TiO_2_NPs-S and TiO_2_NPs-W; (iii) differences in bio-persistence between NPs shapes were clearly seen through the time exposure; (iv) TiO_2_NPs-W apparently impaired the barrier structure readily after 24 h of NPs treatment; (v) the three shapes of TiO_2_NPs were able to cross the mucus shed, enter into the cell’s cytoplasm and, finally, go close the cell nucleus; (vi) more TiO_2_NPs-R/cell nucleus interaction events were seen when compared to TiO_2_NPs-S and -W, at the same concentration; and (vii) TiO_2_NPs-W presented more paracellular transport through the barrier than the other shapes. Taken together, we demonstrate the usefulness of our methodological approach, as well as the differential uptake depending on the NPs’ shape. In fact, Chen et al. [45] already demonstrated that spherical TiO_2_NPs and nanorods could be more readily internalized in HeLa cells than filamentous ones. In spite of the obtained images, a limitation of the technique is that it does not differentiate the NPs internalization rate on each cell type used in the model (absorptive or goblet cells). However, both cell types in monocultures have demonstrated to internalize TiO_2_NPs [44, 46].

TiO_2_NPs are absorbed in the gastrointestinal tract, delivered to the bloodstream and distributed to different organs [15]. According to this, translocation through our barrier model must be demonstrated. Using confocal microscopy, we detected the presence of the three different shapes of TiO_2_NPs in the basolateral chamber, indicating the ability of TiO_2_NPs to pass through the *in vitro* intestinal barrier, independently of their shape. A relationship between TiO_2_NPs’ shape-translocation ability was observed with an increasing concentration-dependent translocation for TiO_2_NPs-R and the opposite for TiO_2_NPs-W. Moreover, constant translocation was seen for all TiO_2_NPs shapes, as the amount of NPs was higher at 48 than at 24 h. This leads us to think that the physicochemical characteristics of each TiO_2_NPs shape can influence their translocation rate. Results from Brun et al. [39] only found TiO_2_NPs translocation in a Caco-2/Raji-B model, but not in the Caco-2/HT29-MTX one, suggesting that TiO_2_NPs pass only through transcellular transport through M cells. However, we provided enough evidence that TiO_2_NPs-S, −R and -W can alter the barrier’s integrity and paracellular transport permeability, to translocate to the serosa of the intestinal tract. Moreover, we also observed a clear TiO_2_NPs-S and -R internalization, probably by both cell types, also indicating a putative transcellular transport.

Among the potentially adverse health effects of TiO_2_NPs internalization, many authors point out genotoxicity on target cells. However, previous *in vitro* and *in vivo* studies evaluating the genotoxicity of TiO_2_NPs present conflicting results [47]. The review of Chen et al. [45] stated that *in vitro* models generated more positive results than the *in vivo* ones, and assays detecting DNA and chromosome damage produced more positive outcomes than those measuring gene mutation. Strong evidence indicates that the genotoxicity of TiO_2_NPs is specifically mediated through the generation of oxidative stress [48]. ROS production might lead to the formation of oxidative DNA damage, primarily 8-oxo-dG adducts, which are considered promutagenic lesions [49]. Hence, the accumulation of these lesions could trigger the cell transformation upon chronic exposures [24].

The morphological status of Caco-2 cells can play an important role in genetic damage prevention, as most studies on undifferentiated cells respond to NPs exposure [11, 50, 51], opposite to what is observed in differentiated cells [12]. This was also indirectly measured by the induction of ROS, observed only in undifferentiated Caco-2 cells [44]. This different behaviour can be explained by the higher uptake of NPs observed in undifferentiated cells, when compared to differentiated cells structured as a monolayer, something that has been demonstrated for nanoceria [52]. In our model, none of the tested TiO_2_NPs shapes was able to induce oxidative DNA damage at any of the tested conditions. This would agree with recently reported data in a Caco-2/HT29-MTX model under acute or chronic exposures [38]. In spite of this, we were able to detect DNA strand breaks in TiO_2_NPs-exposed cells. These DNA breaks could result from the direct interaction of TiO_2_NPs with the nucleus, as detected in our confocal images. General DNA strand breaks were detected readily after 24 h in all TiO_2_NPs shapes exposures. Interestingly, the genotoxic damage persisted after 48 h of TiO_2_-R treatment, which were the more biopersistent TiO_2_NPs in our intestinal barrier model. As previously mentioned, both Caco-2 and HT29 cell types may present different cell uptake rates and, consequently, different DNA damage levels. Unfortunately, we were not able to distinguish the most damaged cell type in the pool of Caco-2/HT29 cells, as the techniques used do not permit the identification of a particular cell type. The separation of both populations after the barrier’s exposure to NPs, by cell sorting methodologies, could bring light to this issue.

## Conclusions

As a summary, the results of this study demonstrate that the three different shapes of TiO_2_NPs behave distinctly when dispersed in DMEM cell culture medium. This can affect their agglomeration status and, as a consequence, their toxic effects. The observed toxic effects aggravated by increasing the time of exposure, as a slight but significant reduction in cell viability was seen after 48 h. We have shown that all three NPs were able to cross the mucus layer, the cell barrier model, and reach the basolateral compartment. Both TiO_2_ nanospheres and nanorods were uptaken easier and faster than nanowires, using transcellular transport to cross the barrier model. However, more adverse effects were seen after exposing the barrier to TiO_2_NPs-W, as the nanowires clearly impaired and compromised the Caco-2/HT29 barrier’s integrity and permeability, using the paracellular transport to cross the barrier. Interactions between the three NPs and the cell nuclei were seen by confocal microscopy, and the consequences were reflected in a significant increase in DNA damage levels. However, we cannot discern if each cell type, Caco-2 and HT29, is equally sensitive to the adverse effects of the selected NPs. Although we have only focused on shape and exposure variables we are aware that other factors such as crystallinity can also to have some influence on the behavior of TiO_2_NPs. In fact, the anatase form induced strongly dendritic cells maturation and showed a stronger adjuvant activity in an *in vivo* allergy model than rutile form [53]. Further improvements in the model would be useful to solve some unanswered questions regarding the different sensitivities of the cell components of this model, as well as the effects of long-term exposures. In fact, in Caco-2 monolayers, significant differences between exposures lasting 24 or 72 h have been reported [54]. Nevertheless, in the Caco-2/HT29 model 72 h after barrier differentiation (21 days) the barrier starts to detach and destabilize. This is a challenge that must be overcome.

## Additional file

Additional file 1: **Table S1.** Primer sequences. **Table S2.** Interconversion of the used concentrations. The relationships between μg/mL and μg/cm^2^ are indicated. **Figure S1.** Monolayer confirmation of the intestinal *in vitro* model, Caco-2/HT29. Transversal cuts of the Caco-2/HT29 barrier stained in Alcian Blue (A and C). Transversal cuts of Caco-2 monocultures (B and D) stained in Hematoxylin and Eosin. **Figure S2.** Dynamic light scattering characterization of the NP’s over the incubation time. Hydrodynamic size for TiO_2_NPs-S (A), TiO_2_NPs-R (B), and TiO_2_NPs-W (C), suspended in DMEM cell culture medium at concentrations ranging from 12.5 to 350 μg/mL. Bars that do not share any letter are significantly different according to the one-way ANOVA with a Tukey’s post-test (*P* < 0.05). Data is represented as mean ± SD. **Figure S3.** Three-dimensional confocal image of the Caco-2/HT29 co-culture exposed to 150 μg/mL of TiO_2_NPs-Wires. Cell nuclei (blue) were stained with Hoechst and mucus (red) stained with WGA. NPs were visualized by reflection and marked with a green mask. NPs-cell nucleus interactions are indicated with white circles. Images were processed with the Imaris 7.2.1 software. **Figure S4.** Confocal images of the reflected NPs found in the collected basolateral medium after exposing the Caco-2/HT29 co-culture barrier to 150 μg/mL of TiO_2_NPs. (DOCX 3832 kb)
