# Supplementary material for: Effects of differently shaped TiO2NPs (nanospheres, nanorods and nanowires) on the in vitro model (Caco-2/HT29) of the intestinal barrier
Source: Part Fibre Toxicol. 2018 Aug 7;15:33. doi: 10.1186/s12989-018-0269-x (PMC6081908; doi:10.1186/s12989-018-0269-x)
Supplement: Supplementary file 1 — Table S1. Primer sequences. Table S2. Interconversion of the used concentrations. The relationships between μg/mL and μg/cm2 are indicated. Figure S1. Monolayer confirmation of the intestinal in vitro model, Caco-2/HT29. Transversal cuts of the Caco-2/HT29 barrier stained in Alcian Blue (A and C). Transversal cuts of Caco-2 monocultures (B and D) stained in Hematoxylin and Eosin. Figure S2. Dynamic light scattering characterization of the NP’s over the incubation time. Hydrodynamic size for TiO2NPs-S (A), TiO2NPs-R (B), and TiO2NPs-W (C), suspended in DMEM cell culture medium at concentrations ranging from 12.5 to 350 μg/mL. Bars that do not share any letter are significantly different according to the one-way ANOVA with a Tukey’s post-test (P < 0.05). Data is represented as mean ± SD. Figure S3. Three-dimensional confocal image of the Caco-2/HT29 co-culture exposed to 150 μg/mL of TiO2NPs-Wires. Cell nuclei (blue) were stained with Hoechst and mucus (red) stained with WGA. NPs were visualized by reflection and marked with a green mask. NPs-cell nucleus interactions are indicated with white circles. Images were processed with the Imaris 7.2.1 software. Figure S4. Confocal images of the reflected NPs found in the collected basolateral medium after exposing the Caco-2/HT29 co-culture barrier to 150 μg/mL of TiO2NPs. (DOCX 3832 kb) [file 12989_2018_269_MOESM1_ESM.docx]

**SUPPLEMENTARY DATA**

**Table S1.** Primer sequences

| **Gene** | **Primer Sequence** |
| --- | --- |
| **SLC15A1** (Forward) | 5’-CTTCGATGCTGTGCTGTACC-3’ |
| **SLC15A1** (Reverse) | 5’-GGCCAAGTGTCACCATCTCT-3’ |
| **SI** (Forward) | 5’-TGGTGGCACTGTTATCCGAC-3’ |
| **SI** (Reverse) | 5’-GACCACCACGGACATGTAGG-3’ |
| **ALPI** (Forward) | 5’-GTCCATCCTGTACGGCAATG-3’ |
| **ALPI** (Reverse) | 5’-ACATGCGCTACGAAGCTCTG-3’ |
| **CLDN2** (Forward) | 5’-TACTCACCACTGGTGCCTGA-3’ |
| **CLDN2** (Reverse) | 5’-GAGAGCTCCTTGTGGCAAGA-3’ |
| **OCLN** (Forward) | 5’-ACAGACTACACAACTGGCGG-3’ |
| **OCLN** (Reverse) | 5’-GCAGCAGCCATGTACTCTTC-3’ |
| **ZO1** (Forward) | 5’-GAGAGGTGTTCCGTGTTGTG-3’ |
| **ZO1** (Reverse) | 5’-GCTGCGAAGACCTCTGAATC-3’ |
| **Actin** (Forward) | 5’-GCATGGAGTCCTGTGGCATC-3’ |
| **Actin** (Reverse) | 5’-CCACACGGAGTACTTGCGCT- 3’ |

**Table S2.** Interconversion of the used concentrations. The relationships between µg/mL and µg/cm^2^ are indicated

| TiO_2_NPs Concentrations | | | | | | |
| --- | --- | --- | --- | --- | --- | --- |
| µg/mL | 12.5 | 25 | 50 | 100 | 150 | 350 |
| µg/cm^2^ | 5.58 | 11.16 | 22.32 | 44.64 | 66.96 | 156.26 |

**FIGURE LEGEND**

**Figure S1**. Monolayer confirmation of the intestinal *in vitro* model, Caco-2/HT29. Transversal cuts of the Caco-2/HT29 barrier stained in Alcian Blue (A and C). Transversal cuts of Caco-2 monocultures (B and D) stained in Hematoxylin and Eosin.

**Figure S2**. Dynamic light scattering characterization of the NP’s over the incubation time. Hydrodynamic size for TiO_2_NPs-S (A), TiO_2_NPs-R (B), and TiO_2_NPs-W (C), suspended in DMEM cell culture medium at concentrations ranging from 12.5 to 350 μg/mL. Bars that do not share any letter are significantly different according to the one-way ANOVA with a Tukey's post-test (*P*<0.05). Data is represented as mean ± SD.

**Figure S3**. Three-dimensional confocal image of the Caco-2/HT29 co-culture exposed to 150 μg/mL of TiO_2_NPs-Wires. Cell nuclei (blue) were stained with Hoechst and mucus (red) stained with WGA. NPs were visualized by reflection and marked with a green mask. NPs-cell nucleus interactions are indicated with white circles. Images were processed with the Imaris 7.2.1 software.

**Figure S4**. Confocal images of the reflected NPs found in the collected basolateral medium after exposing the Caco-2/HT29 co-culture barrier to 150 µg/mL of TiO_2_NPs.

.

**Figure S1.**


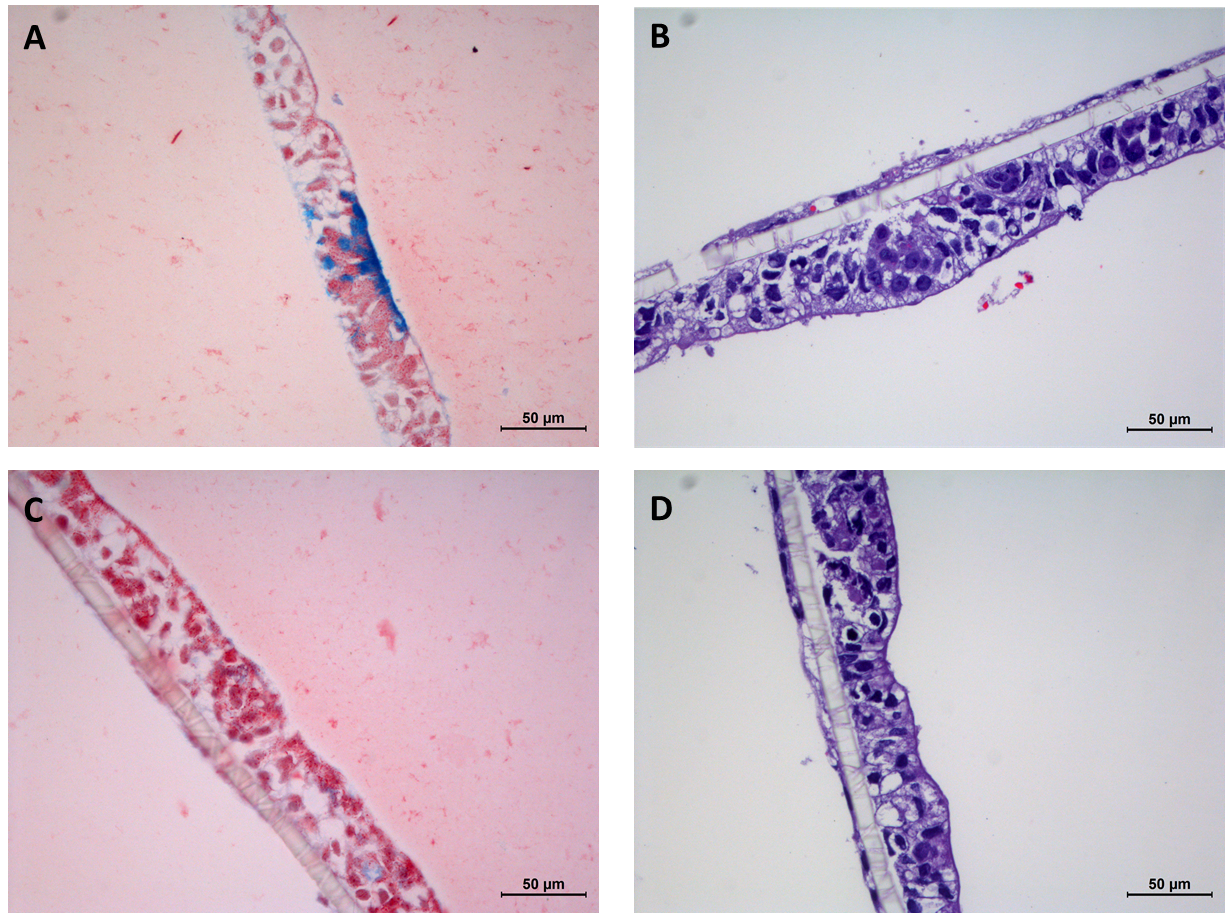


**Figure S2.**

**Figure S3.**

**
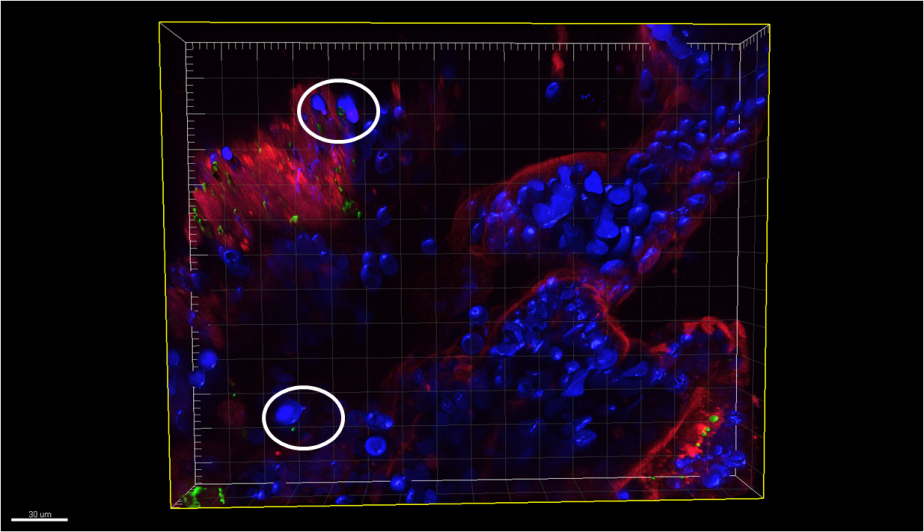
**

**Figure S4.**

**
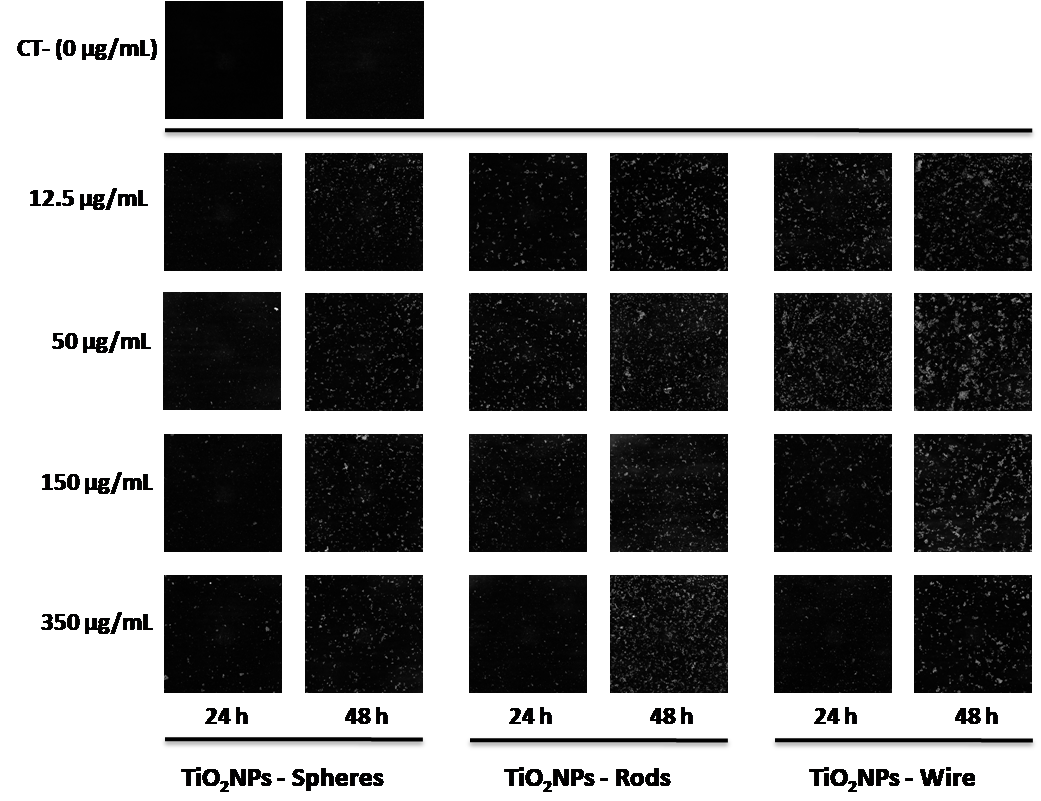
**
